# Longitudinal Assessment Using Optical Coherence Tomography in Patients with Friedreich’s Ataxia

**DOI:** 10.3390/tomography7040076

**Published:** 2021-12-08

**Authors:** Petya Bogdanova-Mihaylova, Helena Maria Plapp, Hongying Chen, Anne Early, Lorraine Cassidy, Richard A. Walsh, Sinéad M. Murphy

**Affiliations:** 1 National Ataxia Clinic, Department of Neurology, Tallaght University Hospital, Tallaght, Dublin 24, Ireland; Richard.Walsh@tuh.ie (R.A.W.); Sinead.Murphy@tuh.ie (S.M.M.); 2 School of Medicine, Trinity College Dublin, Dublin 2, Ireland; plapph@tcd.ie (H.M.P.); chenho@tcd.ie (H.C.); 3 Department of Ophthalmology, Tallaght University Hospital, Dublin 24, Ireland; dr.anne.early@gmail.com (A.E.); Lorrainecassidy23@icloud.com (L.C.); 4 Dublin Neurological Institute at the Mater Hospital and University College Dublin, Dublin 7, Ireland; 5 Academic Unit of Neurology, Trinity College Dublin, Dublin 2, Ireland

**Keywords:** Friedreich’s ataxia, FRDA, optical coherence tomography, OCT, retinal nerve fibre layer, RNFL

## Abstract

Ocular abnormalities occur frequently in Friedreich’s ataxia (FRDA), although visual symptoms are not always reported. We evaluated a cohort of patients with FRDA to characterise the clinical phenotype and optic nerve findings as detected with optical coherence tomography (OCT). A total of 48 patients from 42 unrelated families were recruited. Mean age at onset was 13.8 years (range 4–40), mean disease duration 19.5 years (range 5–43), mean disease severity as quantified with the Scale for the Assessment and Rating of Ataxia 22/40 (range 4.5–38). All patients displayed variable ataxia and two-thirds had ocular abnormalities. Statistically significant thinning of average retinal nerve fibre layer (RNFL) and thinning in all but the temporal quadrant compared to controls was demonstrated on OCT. Significant RNFL and macular thinning was documented over time in 20 individuals. Disease severity and visual acuity were correlated with RNFL and macular thickness, but no association was found with disease duration. Our results highlight that FDRA is associated with subclinical optic neuropathy. This is the largest longitudinal study of OCT findings in FRDA to date, demonstrating progressive RNFL thickness decline, suggesting that RNFL thickness as measured by OCT has the potential to become a quantifiable biomarker for the evaluation of disease progression in FRDA.

## 1. Introduction

Friedreich’s ataxia (FRDA) is the most frequent autosomal recessive (AR) inherited ataxia [1,2], caused by a defect in the frataxin (*FXN*) gene on chromosome 9 (9q13-q21.1,57) [3]. The vast majority of affected individuals are homozygous for a GAA triplet repeat expansion in intron 1 [3], while the remaining patients harbour the GAA expansion on one allele and a point mutation or deletion on the other [3,4,5]. All genetic abnormalities in *FXN* lead to a significantly decreased level of the nuclear-encoded mitochondrial protein frataxin, resulting in the clinical picture of progressive limb and gait ataxia, proprioceptive loss, areflexia, dysarthria, visual dysfunction and hearing loss [6,7,8,9], commonly associated with cardiomyopathy, diabetes and skeletal deformities [7,8,10,11,12].

Ocular motor abnormalities, reflecting the disruption of the brainstem-cerebellar circuits [13,14], are well characterised in FRDA. The most common feature is fixation instability, interrupted by involuntary saccades, or square-wave jerks (SWJ) [13,14,15,16]. Nystagmus is less common but still frequent, and other abnormalities include ocular flutter, saccadic dysmetria, disrupted pursuits and symptomatic oscillopsia [8,10,13,15,17]. Early, primarily descriptive studies of the afferent visual system in FRDA reported optic disc pallor on fundus examination, documented in 30% of affected individuals [10]. With advances in ocular imaging techniques, involvement of the afferent visual pathways in FRDA has been further investigated. A handful of studies evaluated peripapillary retinal nerve fibre layer (RNFL) changes using optical coherence tomography (OCT) [18,19,20,21,22] and reported decreased average RNFL thickness [20], which was statistically significant in comparison to controls [18,19,21,22]. While abnormal RNFL thinning in all quadrants has been reported [18,21,22], a distinctive pattern with predominantly superior quadrant RNFL involvement was observed in one study [18]. In contrast to the RNFL thickness changes, macular and foveal thickness findings detected with OCT in FRDA cohorts are less consistent [18,20,21,22]. Recently, FRDA was reported to be associated with the greatest degree of RNFL thinning in comparison to a range of other genetically characterised ataxias [23].

RNFL thickness in FRDA has been shown to correlate with neurological function and disability as measured with the International Cooperative Ataxia Rating Scale (ICARS) [19,20,21], although not consistently with the Friedreich’s Ataxia Rating Scale (FARS) [18,20]. More recently, a correlation between the peripapillary RNFL and the Scale for Assessment and Rating of Ataxia (SARA) [24] used to quantify disability was demonstrated by Parkinson et al. and Rojas et al. [22,25].

To date, only one small study of eight FRDA patients evaluated RNFL changes longitudinally over a period of six months [22]. No larger studies have been performed to further characterise progressive RNFL thickness changes and their relationship with disease severity.

The aim of this study was to characterise clinical and retinal findings in Irish patients with FRDA. Furthermore, we sought to compare RNFL measurements as detected by OCT to age- and sex-matched healthy controls; to evaluate if a correlation could be found between OCT measurements as an anatomical marker and both disease severity quantified with SARA and disease duration; to document retinal changes detected by OCT over time and determine the usefulness of OCT as an imaging biomarker of disease progression in a larger cohort of FRDA patients.

## 2. Materials and Methods

A total of 48 adult patients were recruited from the Irish National Ataxia Clinic in Tallaght University Hospital, Dublin. Also, 48 healthy controls of comparable age and sex, and without evidence of either optic disc or retinal disease were recruited from the community, as well as from relatives that were not genetically related to recruited patients. Written consent was obtained from all participants before inclusion in the study, which was approved by the joint Tallaght University Hospital and St. James’ Hospital Research Ethics committee.

All affected individuals had a confirmed molecular diagnosis of FRDA through commercially available testing. The majority of patients had two expanded GAA alleles, while one individual was compound heterozygous for a GAA repeat expansion and a point mutation [5].

All patients underwent clinical assessment using a standardised approach, including a detailed history with the age of symptom onset, disease duration, pedigree and comprehensive neurological examination performed by a neurologist.

The Scale for the Assessment and Rating of Ataxia (SARA), consisting of eight quantitative examination features for gait, stance, sitting, speech disturbance and limb kinetic functions, and yielding a composite ataxia score in the range of 0 (no ataxia) to 40 (most severe ataxia) [24] was obtained for each affected individual.

The best-corrected visual acuity (BCVA) was measured from a standard distance of 20 feet using Snellen eye chart with a white background; results were expressed in the logarithm of the minimal angle of resolution (LogMAR) for analysis.

Each participant had a spectral-domain OCT examination with Topcon 3D OCT-2000 (Topcon, Tokyo, Japan), performed in a darkened room. Images were acquired in the seated position with the subjects facing the OCT equipment. After registering the participant, a scanning pattern from the custom options available was selected. Throughout scanning, participants were instructed to fixate on an internal green target provided by the equipment.

Both eyes of each participant were scanned using two standard acquisition protocols with a scanned area of 6-mm cube: macular (6.0 × 6.0 mm, 512 × 128) and 3D optic disc (6.0 × 6.0 mm, 512 × 128). In subjects with involuntary eye movements repeated scans were required in order to obtain images without eye movement artefacts.

In a small number of cases, imaging of both eyes was not possible due to technical reasons, including eye movement artefacts or failure to achieve acceptable image quality. Scans with poor image quality, defined as scans with an image quality score of ≤50/100 were excluded from the analysis.

In each case, results were evaluated after the examination was complete.

For the RNFL analysis, an optic disc cube of data centred in the optic nerve head was acquired. Evaluated peripapillary RNFL thickness parameters included average thickness of a 360° measurement, and the thickness in each quadrant around the disc: superior (46° to 135°), nasal (136° to 225°), inferior (226° to 315°) and temporal (316° to 45°) for the right eye; superior (46° to 135°), temporal (136° to 225°), inferior (226° to 315°) and nasal (316° to 45°) for the left eye. Evaluated macula thickness parameters included overall thickness and foveal thickness. According to the Early Treatment Diabetic Retinopathy Study (ETDRS) grid [26], the macula is divided into nine regions with three concentric rings measuring 1 mm (innermost ring), 3 mm (inner ring) and 6 mm in diameter (outer ring) centred on the fovea; the 3 mm inner ring and 6 mm outer ring are further divided into four equal grids. The foveal thickness was defined as macular thickness within the innermost 1 mm ring of the ETDRS map, while mean macular thickness was defined as the average macular thickness from all nine regions of the ETDRS map.

Following scan acquisition, the macular and RNFL measurements were automatically calculated by OCT using the existing software.

Normative data for the Spectral-Domain OCT examination with Topcon 3D OCT-2000 (SD-OCT) were provided by a study of 189 healthy individuals with an age range between 19–84 years [27].

A follow-up assessment was performed on 20 individuals. All tests were performed by the same examiner under the same conditions at baseline and at follow-up visits.

Statistical analysis was performed using Microsoft Excel (2013) and IBM^®^ SPSS^®^ Statistics for Windows version 25.0.

A nonparametric Mann–Whitney U test was used to compare demographic differences between patients and controls. For group quantitative analysis, a mixed linear analysis was used. RNFL parameters and foveal thickness were correlated with the SARA and visual acuity using Pearson’s correlation test, while the relationship between the OCT parameters and disease duration was analysed using Spearman’s rank correlation test.

Changes in RNFL thickness from baseline were assessed in 20 patients who had at least one follow-up visit using also a mixed linear analysis using the time as a variable and accounting for expected minimal age-related RNFL thinning [28].

A *p*-value <0.05 was considered statistically significant in all analyses.

## 3. Results

### 3.1. Cohort Description

A total of 48 Caucasian affected individuals from 42 unrelated families, all of Irish descent, were included. There was a slight predominance of males (n = 26, 54.2%).

Of these, 26 individuals (54.2%) had no relevant family history. The remaining 22 cases (43.8%) were familial with inheritance compatible with an autosomal recessive mode in all but one family (patient 18, Table 1), where a pseudo-dominant pattern with disease transmission from one generation to the next was observed. In this family, the mother and a number of siblings were affected, while the father was a carrier.

The age-at-symptom-onset ranged between 4–40 years (mean 13.8 ± 8.1), while disease duration varied between 5–43 years (mean 19.5 ± 9.9). The mean age at assessment was 33.4 ± 13 years (range 18–63).

The demographic and clinical data of FRDA patients are shown in Table 1.

In all affected FRDA individuals, gait instability was a presenting symptom. All patients displayed variable degrees of ataxia and the majority (36/48, 75%) required a wheelchair for mobilising with usage ranging from occasional to all the time. Cardiomyopathy in its hypertrophic form was documented with an echocardiogram in 21/48 (43.8%), while 10 individuals (20.8%) had diabetes. A small proportion, 6/48 (12.5%), had hearing loss and of the 18 individuals with scoliosis 10 patients had prior surgery (37.5%).

SARA was highly variable and ranged between 4.5–38/40 (mean 21.9/40 ± 8.5).

### 3.2. Ophthalmological Findings

Mean BCVA in FRDA patients was significantly lower compared to controls (*p* < 0.05); mean LogMar in the patient group was 0.26 ± 0.5 (range 0 to 3.0) vs. 0.03 ± 0.07 (range −0.0 to 0.3) in controls. In 30/48 patients (62.5%), LogMar VA was 0.2 or better.

The majority of FRDA patients (n = 32, 66.7%) had abnormal ocular motor findings (Table 2). Square wave jerks (SWJ) were documented in 28/48 (58%), while one individual had an ocular flutter. Less than half of individuals with FRDA had nystagmus (20/48, 41.7%).

**Figure 1:**
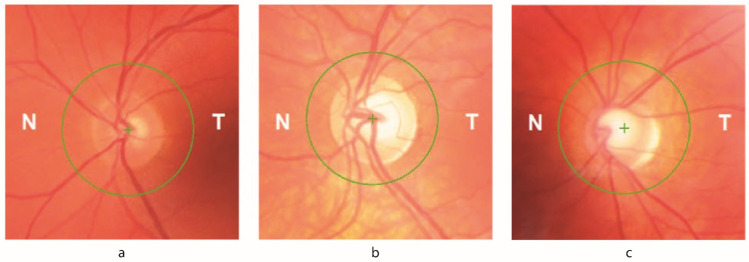
Retinal fundus images with circular volume scan delineation of FRDA patients with varying degrees of optic neuropathy. (**a**) Normal appearance of the optic disc (patient 33); (**b**) Optic disc pallor in a patient with normal visual acuity (patient 2); (**c**) Diffuse optic disc pallor in a patient with reduced visual acuity (patient 21). N Nasal; T Temporal.

Fundus examination revealed variable optic disc features ranging from essentially normal appearance to diffuse optic disc pallor; in 21/48 (43.8%) of patients, a variable degree of optic disc pallor was documented (Figure 1).

### 3.3. Optical Coherence Tomography

OCT data from all 48 FRDA were compared with those of 48 healthy controls (23 males; mean age at assessment 33.9 ± 10.2 years, range 16–63). There was no difference between patients and controls in terms of gender (*p* = 0.7) and age (*p* = 0.5).

In total four eyes were excluded from macular analysis due to poor image quality as a result of severe visual loss or fixation instability. Valid RNFL parameters data were obtained from 69 eyes.

The results of OCT studies are summarised in Table 2.

There was a statistically significant reduction in the average peripapillary RNFL thickness, as well as in all sectors except for the temporal in the patient group in comparison to controls (Table 3). The average macular and foveal thicknesses were also significantly reduced in patients, compared to controls.

**Figure 2:**
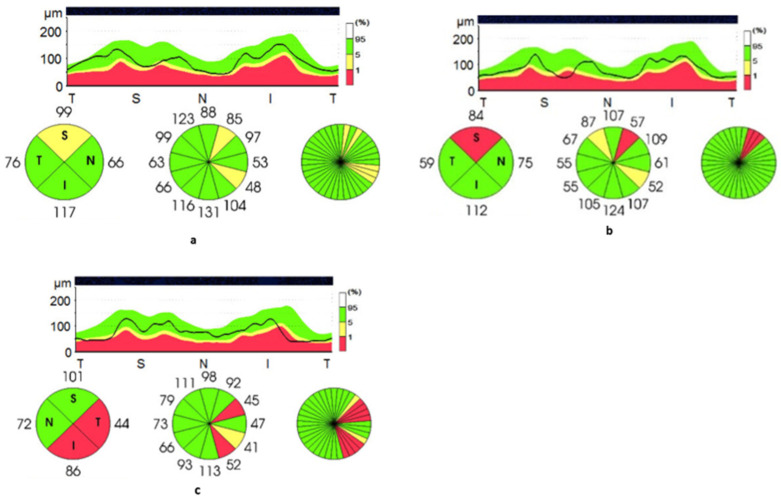
Retinal nerve fibre layer thickness of patients with FRDA. This figure demonstrates reduced sectorial superior quadrant thickness in patients 42 (**a**) and 5 (**b**), and reduced temporal and inferior quadrant thickness in patient 45 (**c**). Pie graphs of quadrants (T = temporal; S = superior; N = nasal; I = inferior) and RNFL circular tomogram representing quantitative analysis of RNFL thickness (black line) and normative data set (green area = 95% confidence interval, yellow area = 99% CI, red area = outside 99% CI).

Of the affected individuals, 43.6% (17/39) had superior quadrant thickness below the 95% lower limit of normal, defined in the normative database provided by the manufacturer, for example, as shown in Figure 2a,b. Furthermore, 28.2% (11/39) had inferior quadrant reduction below the 95% lower limit, while nasal and temporal thickness reduction below the 95% lower limit of normal was documented in 5 and 4 individuals, respectively. The OCT of the individual with a GAA repeat expansion on one allele and a point mutation on the other allele showed significant sectorial RNFL thinning below the normal limits both in the temporal and inferior quadrants (Figure 2c).

### 3.4. Correlation with Clinical Features

There was a significant correlation between baseline macular, average peripapillary and RNFL thickness in all sectors but the nasal, and disease severity as quantified with SARA (Supplementary Table S1). Visual acuity was correlated with the average macular and RNFL thicknesses. No significant association was found between disease duration and RNFL thickness, but the relationship trended towards significance for the macular thickness.

Relationships between the OCT parameters and disease duration were analysed using the Spearman’s rank correlation test; relationships between the OCT parameters and disease severity as quantified with SARA, and visual acuity were analysed using Pearson’s correlation.

### 3.5. Longitudinal Assessment

In total, 20 individuals had repeated OCT studies during a mean follow-up interval of 28.4 months (range 12–48). Only macular OCT images from one individual (patient 29) were analysed due to poor image quality at both assessments.

**Figure 3:**
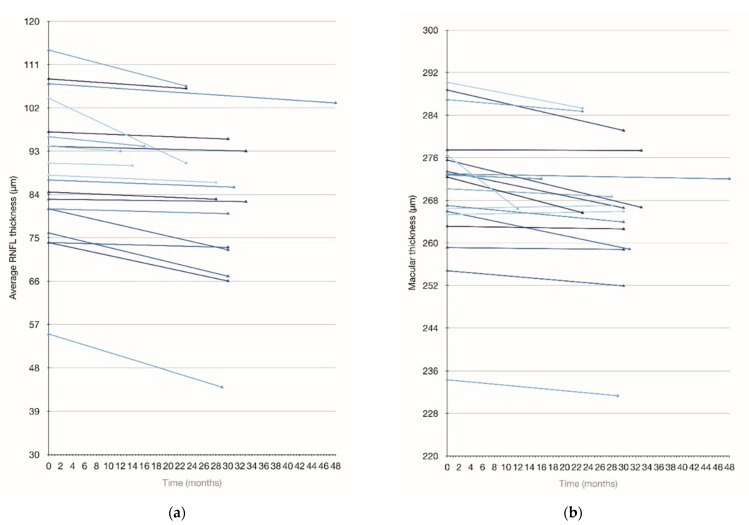

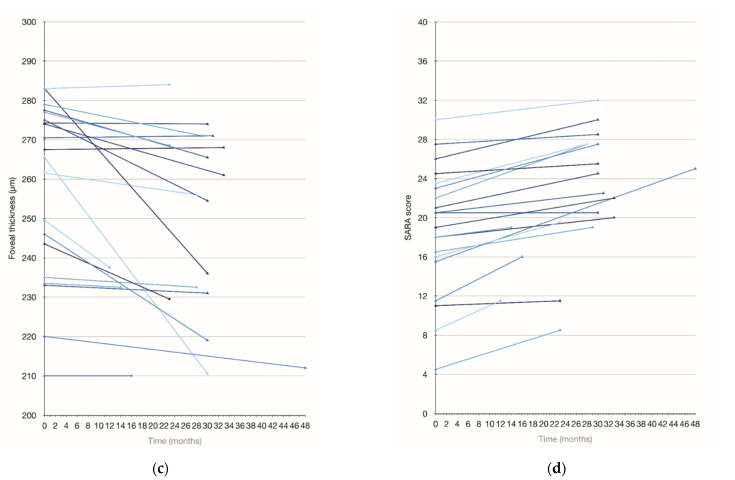
Retinal thickness and SARA score over time. (**a**) Average RNFL thickness at baseline and at follow-up evaluation; (**b**) Macular thickness at baseline and at follow-up evaluation; (**c**) Foveal thickness at baseline and at follow-up evaluation; (**d**) SARA score at baseline and at follow-up assessment.

There was a significant decline in the average peripapillary RNFL thickness (Figure 3a), as well as in most of the RNFL sectors over time (Table 4 and Supplementary Table S2). Statistically significant thinning was also documented in the average macular thickness (Figure 3b) and fovea (Figure 3c). Disability progression, documented in 19/20 patients, is shown in Figure 3d (mean SARA at baseline 18.9 ± 6.5, at follow-up 21.9 ± 6.5, *p* < *0*.05).

At follow-up assessment, significant correlation between RNFL thickness and disease severity as quantified by SARA was found once more (r_Average RNFL_ vs. _SARA_= −0.467, *p* = 0.04). Similar to the baseline assessment, there was no association between disease duration and RNFL or macular thickness (r_Average RNFL_ vs. _duration_ = −0.291, *p* = 0.22; r_Macula_ vs. _duration_ = −0.059, *p* = 0.8).

## 4. Discussion

This is the first Irish study investigating retinal involvement in individuals with FRDA using OCT. Our results confirm significant RNFL and macular thinning even in patients without clinically apparent visual impairment and highlight the usefulness of OCT in detecting these changes as the disease progresses. A modest number of prior OCT studies evaluated retinal changes in FRDA and the size of our cohort compares with the two largest published to date: 57 and 52 individuals in the US and UK studies, respectively [18,25]. Similar to an Italian study in which 80% of the 26 FRDA patients were visually asymptomatic but all had evidence of underlying optic neuropathy detected with OCT [19], the majority of patients in our cohort (~70%) exhibited some degree of afferent visual pathway involvement, apparent clinically only in a small proportion of affected individuals.

In this non-homogeneous FRDA cohort, both functional (BCVA) and structural (OCT) visual measures correlated with neurological disability as quantified with SARA, indicating that these can potentially serve as functional and anatomic measures of disease progression in future clinical trials. Similar to our results, Seyer et al. [18] reported correlations between OCT parameters and neurologic function as measured by FARS, which were slightly higher than associations with visual functions, proposing that OCT may ultimately be applicable as an anatomic biomarker of structural neuronal loss in FRDA.

The significant reduction in average peripapillary RNFL thickness found in this FRDA cohort is consistent with previous reports [18,19,20,21,22,23,25]. In contrast to other nuclear mitochondrial disorders where the temporal quadrant thickness is most affected [29], temporal RNFL thickness in our cohort was least decreased, similar to previously reported observations in FRDA [20]. Moreover, close to half of our patients had superior quadrant involvement, reported as the predominantly affected sector in FRDA by Seyer et al. [18]. Notably, when compared with individuals with other genetically confirmed subtypes of ataxia evaluated in our clinic, including *SPG7*-associated spastic ataxia, autosomal recessive spastic ataxia of Charlevoix-Saguenay (ARSACS), common spinocerebellar ataxias (SCAs), cerebellar ataxia with neuropathy and vestibular areflexia syndrome (CANVAS), and *AIFM1*- associated phenotype among others [29,30,31,32] (and unpublished data) the peripapillary RNFL thickness in the FRDA cohort was significantly thinner. Our OCT results are comparable with one previous study where, among genetically determined inherited ataxias, FRDA was associated with the most notable RNFL thinning [23] and support the potential value of OCT in distinguishing FRDA from other progressive ataxias.

The classical optic neuropathy of mitochondrial dysfunction is associated with a marked preference for damage to the papillo-macular bundle, which contains the highest density of retinal ganglion cells (RGCs), resulting in central vision loss and temporal pallor of the optic nerve [33,34]. Leber’s hereditary optic neuropathy (LHON) and dominant optic atrophy (DOA), the most common non-syndromic mitochondrial optic neuropathies, share overlapping clinical and pathological features and are characterised by early loss of RGCs within the papillo-macular bundle [33,35]. The selective vulnerability of the optic nerve in classical mitochondrial optic neuropathies has been related to uneven distribution of mitochondria and thus unequal energy demands along each RGC axon [36]. Histochemical studies have shown mitochondrial clustering in areas with a high density of repolarising sodium-potassium membrane pumps, and an abrupt decrease in mitochondrial numbers posterior to the lamina cribrosa where myelination begins and energy-efficient saltatory conduction occurs [33,37,38]. A combination of energy failure, oxidative stress, and predisposition to apoptosis along with mitochondrial distribution within this axonal system is considered the pathological basis for RGC degeneration in both LHON and DOA [39].

In contrast, optic neuropathy observed in FRDA does not follow the classical mitochondrial pattern. The papillo-macular axonal system is not preferentially involved, suggesting that in the mitochondrial disorder associated with a genetic defect in *FXN*, the pattern of visual impairment and the underlying pathological mechanism are different from those in LHON and DOA [19]. The encoded mitochondrial protein frataxin is directed to the mitochondrial inner membrane and is involved in the assembly of iron–sulphur clusters, which are critical components of the mitochondrial respiratory chain complexes [40]. Alterations in frataxin affect the respiratory chain and lead to bioenergetic impairment, increased oxidative stress, and abnormal accumulation of intra-mitochondrial iron, which eventually reaches toxic levels [41], thus increasing the sensitivity of cells to undergoing apoptosis [41,42,43]. Decreased expression of frataxin could cause pathologic changes in restricted groups of tissues and it has been proposed that optic neuropathy in FRDA is a result of the increased sensitivity of RGC to oxidative stress. An impaired cellular defence against reactive oxygen species in FRDA likely further exacerbates neuronal loss [44]. As the cells have some mechanisms for managing reactive oxygen species, the cumulative damage in the retina occurs gradually, leading to visual impairment [45].

By definition, the non-syndromic optic neuropathies are limited to a single cellular target, that is, the RGCs. These seem more specifically related to defective complex I function [33] and involve a more specific subset of visual fibres. In contrast, in FRDA complexes I, II and III are involved which may be limiting the compensatory mechanisms likely existing in LHON and DOA, and as a result, cause less selective damage to RGC populations [19]. Furthermore, the clinical expression in FRDA is associated with much more severe and widespread presentation, behaving as a multi-systemic mitochondrial disorder also affecting the optic nerve. Interestingly, despite different pathological mechanisms involving the visual pathways in FRDA, a subacute or acute visual failure mimicking LHON may develop in the presence of high GAA triplet expansion or in compound heterozygotes, superimposed on the slow progression of FRDA optic neuropathy [19]. Unlike previously reported higher incidence of optic disc pallor in compound heterozygotes than in expansion homozygotes [46], our single compound heterozygous individual was visually asymptomatic and had no disc pallor on fundus examination.

Only a handful of studies have evaluated the macular thickness in FRDA patients. Similar to the US and the small Spanish groups [18,22], macular thickness in our FRDA cohort was reduced compared to controls, in contrast to the larger Spanish study, where the macular thickness was normal, but no comparison to controls was documented [20]. Furthermore, our study found significantly reduced foveal thickness, previously only documented in 10 eyes of 10 Turkish patients [21], but not observed by Noval et al., or Rojas et al. [20,22] and not reported in other studies. A prior electroretinography study did not detect substantial retinal abnormalities in FRDA, suggesting that loss of low-contrast vision and peripheral visual field with preservation of high-contrast vision would be against primary macular disease [47]. However, more recent OCT data suggest that macular changes may be a component of the most advanced visual impairment in FRDA and could clinically affect the vision only in the most severely affected patients [18].

Similar to two other studies, we found a correlation between RNFL thickness and disability as quantified with SARA, demonstrating that as the disease progresses and neurological function deteriorates, the RNFL decreases [22,25]. In prior studies, RNFL thickness was correlated with disability as measured with two other much more extensive ataxia rating scales: ICARS and FARS. While no association was found between RNFL thickness and disease severity as measured by FARS in a study of 23 FRDA patients [20], a correlation was found between RNFL thickness and ICARS, the only scale that considers features not directly related to the physical examination, such as activities of daily living. Three further studies documented correlations between RNFL and disability as quantified with ICARS [19,20] and FARS [18]. Overall, these findings suggest that RNFL thickness might be a useful marker of disease progression in FRDA and that OCT may have an advantageous role in therapeutic trials.

In our study, no correlation was found between RNFL thickness and disease duration, observed in some FRDA cohorts [18,24,25] but not documented in others [19,20]. This discrepancy is possibly due to the highly variable disease duration in our FRDA group in comparison to other cohorts where an association was found, although the number of evaluated subjects here was much higher than in the two cohorts where no correlation was found. Findings from a small number of OCT studies in various SCAs have suggested that RNFL changes are unrelated to disease duration, similarly to our results [48,49]. In all subtypes of CA, the rate of progression varies from person to person. Furthermore, progression rates in different entities are not constant during the long disease duration, e.g., in the early phase of SCA2, progression is slower than in the following years [50]. In contrast, a much faster rate of progression is seen in FRDA, where other factors, such as GAA repeat length among others, determine the speed of progression.

To our knowledge, this is only the second longitudinal OCT study in FRDA patients and is the largest such study to date [22]. Our results demonstrated significant retinal decline and disability progression over time, confirming prior observations in FRDA patients evaluated over a much shorter time interval of 6 months. The interpretation of results in longitudinal OCT studies may be challenging due to the insufficient knowledge regarding the rate of RNFL loss in FRDA. Henderson et al. speculated that RNFL thinning in MS, another degenerative disease of the CNS, is probably not a linear process and that there is a more rapid RNFL loss in the early stages of the disease [51]. In a small longitudinal study of four individuals with SCA3, five out of eight eyes evaluated showed a mild trend to RNFL thinning and the progression rate per year was calculated [49]. However, because of the small sample size and the absence of a control group, it is unclear if the detected loss represented progression of the disease or was attributable to other factors such as variability between exams. In our study, healthy controls were not evaluated at an interval, and thus no comparisons were made. To date, it remains uncertain if there is any RNFL thinning dynamic during the FRDA disease course.

Two previous OCT studies in FRDA cohorts were performed entirely using SD-OCT [21,22]. One of the groups used Time-Domain OCT (TD-OCT) for RNFL evaluation and SD-OCT for the macular analysis [18], while TD-OCT was utilised in the remaining FRDA studies [19,20,25]. Prior evaluation of the intersystem reproducibility showed that there is a discrepancy between results from different OCT devices. In a cross-sectional study of 52 healthy eyes, the macular thickness measurements obtained by SD-OCT devices were higher than those obtained by TD-OCT [52]. Furthermore, RNFL analysis of patients with MS and healthy controls performed with TD- and SD-OCT found a strong correlation, but a statistically significant difference between the two devices, suggesting that measurements between different generations of OCT machines (TD-OCT versus SD-OCT) are not interchangeable. The discrepancy between the measurements obtained with different devices may have particular implications for longitudinal studies if switching OCT machines when monitoring retinal changes over time [53].

The strengths of the study are the sample size, its prospective design and comparisons with matched controls. While this study demonstrates that OCT can be used to measure directly neuronal loss in FRDA, it has several limitations. In a proportion of patients, significant disability and eye movement abnormalities led to missing scans. Another weakness is that we did not use an SD-OCT with layer segmentation analysis, and ganglion cell complex (GCC) thickness was not measured. Individuals included in the longitudinal part of the study were not evaluated at precise time intervals between baseline and follow-up, and the progression rate was not calculated.

A number of potential therapeutics have been tested in clinical trials in FRDA patients, but at present, there is no approved therapy. In other neurodegenerative diseases, the need for disease-modifying treatments has been facilitated by the identification of potential markers that can capture subclinical changes in a rapid manner, and therefore can be useful in clinical trials of slowly progressive neurological disorders. In FRDA, important progress has been made in addressing the various aspects of biomarkers use, including their diagnostic, monitoring, response, predictive and prognostic role [54]. In contrast to previously proposed FRDA biomarkers, OCT is a non-invasive, quick and increasingly available test for identifying characteristic retinal changes in individuals with FRDA. In addition, this technique demonstrates high reproducibility and therefore has the potential to be reliably utilised in future clinical trials.

## 5. Conclusions

This study highlights that FDRA is associated with frequent subclinical optic neuropathy. Our results demonstrate that RNFL thickness as measured by OCT has the potential to become a quantifiable biomarker for the evaluation of disease progression. Furthermore, the longitudinal data showing significant progression rates of retinal damage detectable through OCT support the usefulness of this cost-effective technique as an objective tool in future therapeutic trials.

## Figures and Tables

**Table 1:** Demographic and clinical characteristics of individuals with FRDA.

Pt	Fam Hx	Sex	Age at Onset (yrs)	Age at Exam (yrs)	Disease Duration (yrs)	SARA	Ataxia	NP	WC Bound	Hearing Loss	LVH	Diabetes mellitus	Scoliosis/Surgery
1	No	M	5	31	26	37	+	+	+	+	+	+	+/no surgery
2	No	F	16	19	3	8.5	+	+	-	-	-	-	-
3	No	M	37	42	5	4.5	+	+	-	-	-	-	-
4	Yes	F	11	26	15	23	+	+	+	-	+	-	+/surgery
5	Yes	M	11	25	14	27.5	+	+	+	-	+	-	-
6	Yes	M	19	40	21	19	+	+	+	-	-	-	+/no surgery
7	Yes	F	19	30	11	11	+	+	-	-	-	-	-
8	Yes	F	16	30	14	13	+	+	-	-	-	-	+/no surgery
9	No	F	16	52	36	36	+	+	+	-	-	+	+/surgery
10	No	M	35	47	12	16	+	-	-	-	-	-	-
11	No	M	11	33	22	38	+	+	+	+	+	-	+/no surgery
12	Yes	M	11	41	30	34	+	+	+	-	+	+	+/no surgery
13	No	F	11	18	7	18	+	+	+	-	-	-	+/+
14	No	F	9	35	26	37	+	+	+	-	+	-	-
15	No	M	5	21	16	16.5	+	+	+	-	-	+	+/+
16	Yes	M	30	63	33	15	+	+	+	+	-	+	-
17	No	M	3	27	24	25	+	+	+	-	+	-	+/+
18	Yes	F	18	54	36	15.5	+	+	+	-	-	-	-
19	Yes	M	5	18	13	28.5	+	+	+	-	-	-	+/-
20	Yes	F	13	19	6	11.5	+	+	-	-	+	-	+/-
21	Yes	F	10	24	14	20.5	+	+	+	-	-	-	+/+
22	Yes	F	11	27	16	19.5	+	+	+	-	-	-	+/+
23	No	M	16	35	19	21	+	+	+	-	-	-	+/-
24	No	F	40	60	20	29	+	+	+	-	-	-	-
25	No	F	5	18	13	24.5	+	+	+	-	+	+	-
26	Yes	M	14	54	40	27.5	+	+	+	+	-	+	-
27	Yes	M	23	45	22	28	+	-	+	-	-	-	+/-
28	No	F	4	31	27	26	+	+	+	-	+	+	+/+
29	Yes	F	12	36	24	30	+	+	+	+	+	-	-
30	No	M	12	51	39	37.5	+	+	+	-	+	-	+/+
31	No	M	10	20	10	23	+	+	+	-	+	-	+/-
32	Yes	M	17	47	30	31.5	+	+	+	-	-	+	+/-
33	Yes	M	21	45	24	18	+	+	-	-	-	-	-
34	No	M	13	18	5	11.5	+	+	-	-	+	-	+/-
35	No	F	7	21	14	27	+	+	+	-	-	+	+/-
36	Yes	M	6	22	16	20.5	+	+	+	-	+	-	+/-
37	Yes	F	8	26	18	26	+	+	+	-	+	-	-
38	No	M	8	18	10	29.5	+	+	+	-	+	-	+/-
39	Yes	F	16	51	35	16	+	+	+	-	-	-	-
40	No	F	10	23	13	19	+	+	+	-	+	-	+/-
41	No	M	15	36	21	14	+	+	-	-	-	-	-
42	No	M	10	19	9	18	+	+	+	-	+	-	+/+
43	Yes	F	9	34	25	23.5	+	+	+	-	-	-	+/+
44	No	F	11	29	18	22	+	+	+	-	+	-	+/-
45	Yes	M	10	53	43	24	+	+	+	-	-	-	-
46	No	F	10	21	11	10.5	+	+	-	-	-	-	+/-
47	No	M	6	22	16	10.5	+	+	-	+	+	-	+/-
48	Yes	M	29	44	15	9.5	+	+	-	-	-	-	-

Fam Hx Family history; LVH Left ventricular hypertrophy; WC wheelchair; SARA Scale for the Assessment and Rating of Ataxia; NP Neuropathy.

**Table 2:** Ophthalmological findings and RNFL results in patients with FRDA.

						Retinal Nerve Fibre Layer						
Pt	SWJ	Ny	BCVA		Optic Disc Pallor	Average Macula (µm)	Foveal (µm)	Average (µm)	Superior (µm)	Nasal (µm)	Inferior (µm)	Temporal (µm)
			R	L								
1	+	+	HM	HM	+	234.6	225					
2	+	-	20/20	20/20	+	276.45	249.5	94	110.5	81.5	115	68.5
3	+	+	20/20	20/25	-	286.9	277	114	122	111	136	87
4	+	+	20/40	20/40	+	267	246	80	101.5	63.5	93.5	63
5	+	+	20/40	20/30	-	259.1	277.5	74	93	55	93	57
6	-	-	20/20	20/20	-	275.55	274	94	101.5	80	127.5	67
7	+	-	20/20	20/20	-	272.35	243.5	108	138	86.5	135	74
8	+	-	20/20	20/20	-	276.85	230	98	106	83	121.5	81.5
9	-	-	NLP	20/50	+	252.8	234					
10	+	-	20/40	20/25	-	290.15	283	104	112	110	109	85
11	+	+	20/70	20/70	-	271.6	238					
12	+	+	20/50	20/50	-	265.8	269					
13	-	-	20/20	20/20	+	264.55	243.5	86.5	99	66	117	76
14	+	+	20/200	20/200	+	254.15	225.5	66	75	95	48	48
15	-	+	20/70	20/30	-	234.3	279	55	62	52	56	50
16	-	-	20/20	20/25	-	260.7	223.5	104	132	78	128	79
17	+	-	20/70	20/40	+	266	274.5	69	70	47	67	54
18	-	-	20/20	20/25	-	272.95	220	107	120	99	120.5	84
19	+	+	20/40	20/40	+	290.8	274					
20	-	+	20/20	20/20	-	279.45	268	99.5	119	101.5	107.5	70.5
21	+	+	20/30	20/30	+	254.75	233	81	87.5	58	107	71
22	-	-	20/25	20/20	-	256	243	82.5	80.5	71.5	100	73
23	+	-	20/20	20/20	-	288.7		74	75	69	85	66
24	+	+	20/30	20/40	-	283.75	210	93	110	68.5	103.5	80.5
25	-	-	20/20	20/25	+	263.1	283	97	116	78.5	113.5	79
26	-	+	20/40	20/40	-	261.1						
27	+	-	20/20	20/20	-	288.85	254	94.5	108.5	86.5	119.5	64
28	+	-	20/20	20/20	+	246.95	207	85	97	67	100	74
29	+	+	20/25	20/30	+	265.3	265.5					
30	+	+	CF	CF	-	267.6	290.5	75	85.5	70	97	60.5
31	+	-	20/25	20/20	-	261.65	277					
32	+	-	20/20	20/20	-	255.15	230	79	95	63	98	62
33	-	+	20/20	20/20	-	272.7	233.5	90.5	103.5	75.5	107.5	74.5
34	+	-	20/30	20/30	-	272.8	210	96	108	54	103	55
35	-	-	20/20	20/20	+	274.1	266.5	98.5	110.5	82	116	87
36	+	-	20/70	20/70	+	258.8	271	87	102.5	80	96	45.5
37	+	+	20/30	20/25	+	273.4	275	76	82.5	70.5	93.5	58
38	+	-	20/50	20/30	-	268.35	267					
39	-	-	20/40	20/40	-	291.35	196.5	105.5	132	82	110.5	97
40	+	+	20/20	20/20	+	265	251	86	88.5	75	107	74.5
41	+	-	20/20	20/20	+	278.9	242.5	99	112.5	81	129.5	74.5
42	-	+	20/20	20/20	+	277.45	267.5	83	89.5	71	110.5	61.5
43	-	-	20/20	20/20	+	266.45	261.5	84.5	75	68.5	118.5	76
44	+	-	20/20	20/20	+	270.15	235	88	95	60	117.5	80.5
45	-	-	20/20	20/20	-	259.05	268.5	80.5	104.5	83	82.5	50.5
46	-	-	20/20	20/20	-	255.45	220	94.5	101	77.5	107.5	93
47	+	-	20/40	20/30	+	272.45	253.5	82.5	73	71	117.5	69
48	+	+	20/20	20/25	-	273.5	239	95.5	108	87.5	109.5	76

SWJ Square wave jerks; Ny Nystagmus; BCVA Best corrected visual acuity quantified as Snellen ratio (ft); HM Hand Movements; CF Counting fingers; NLP No light perception; Presented OCT values for each patient are the mean of measurements from both eyes unless it was only possible to obtain a good quality scan from one eye.

**Table 3:** Demographic and ocular characteristics of affected individuals.

					3D Macula		3D Disc				
		M/F Ratio	Age (Years)	BCVA (LogMAR)	Average (µm)	Foveal (µm)	Average RNFL (µm)	Superior (µm)	Nasal (µm)	Inferior (µm)	Temporal (µm)
**FRDA**	**mean ± SD**	26/22	33.4 ± 13	0.26 ± 0.5	268.1 ± 13.2	250.4 ± 24.2	88.4 ± 12.9	97.9 ± 20.1	75.4 ± 15.1	105.7 ± 19.4	70.3 ± 12.7
	**range**		(18–63)	(0–2.3)	(233.9–294.3)	(194–300)	(55–114)	(58–134)	(54–111)	(56–138)	(51–96)
**Controls**	**mean ± SD**	23/25	34.2 ± 10.2	0.03 ± 0.07	273.8 ± 10.9	238.3 ± 22.8	103.9 ± 8.4	122.3 ± 12.9	88.5 ± 10.1	130.6 ± 11.3	74.5 ± 7.8
	**range**		(16–63)	(-0.1–+0.3)	(252–300)	(214–293)	(92–124)	(90–155)	(72–124)	(110–156)	(60–96)
***p* value**		0.6	0.5	**<0.001**	**0.003**	**<0.001**	**<0.001**	**<0.001**	**<0.001**	**<0.001**	0.102

Numbers in **bold** *p*-value < 0.05.

**Table 4:** Analysis of macular and RNFL thickness over time.

	3D Macula		3D Disc				
	Average (µm)	Foveal (µm)	Average RNFL (µm)	Superior (µm)	Nasal (µm)	Inferior (µm)	Temporal (µm)
**Baseline**	270.3 ± 12.4	257.9 ± 22.3	88.8 ± 14.3	97.6 ± 20.6	75.8 ± 17.1	105.4 ± 18.9	68.9 ± 12.5
**Follow up**	267.4 ± 12.4	246.9 ± 24.1	85.2 ± 15.5	92.6 ± 24.4	72.5 ± 14.9	105.1 ± 21.4	67.1 ± 12.2
***p* value**	**0.01**	**0.007**	**0.001**	**0.016**	**0.036**	**0.046**	0.17

Numbers in **bold** *p*-value < 0.05.

## Data Availability

The data that support the findings of this study are available on request from the corresponding author.

## Supplementary Materials

The following are available online at https://www.mdpi.com/article/10.3390/tomography7040076/s1, Table S1: Correlation between OCT parameters and disease severity, disease duration and visual function. Table S2: Baseline, follow-up and expected follow-up RNFL thickness in FRDA patients.
