# Supplementary material for: Longitudinal Assessment Using Optical Coherence Tomography in Patients with Friedreich’s Ataxia
Source: Tomography. 2021 Dec 8;7(4):915–31. doi: 10.3390/tomography7040076 (PMC8706975; doi:10.3390/tomography7040076)
Supplement: Supplementary file 1 [file tomography-07-00076-s001.zip › tomography-1477638-SM.pdf]

Table S1. Correlation between OCT parameters and disease severity, disease duration and visual function

	Macula		RNFL				
	**Average**	**Foveal**	**Average**	**Superior**	**Nasal**	**Inferior**	**Temporal**
**SARA**	r(47)=–0.320	r(45)=0.080	r(38)=–0.56	r(38)=–0.42	r(38)= –0.308	r(38)=–0.520	r(38)=–0.399
	**p=0.03**	p=0.6	**p=0.0002**	**p=0.008**	p=0.06	**p=0.0008**	**p=0.01**
**Disease duration**	rs = –0.286	rs = –0.259	rs = –0.266	rs = –0.165	rs = –0.057	rs = –0.195	rs = –0.137
	p=0.051	p=0.09	p=0.1	p=0.3	p=0.7	p=0.2	p=0.4
**BCVA**	r(47)= –0.391	r(45=)–0.015	r(38)=–0.396	r(38)=–0.292	r(38)= –0.089	r(38)=–0.44	r(38)=–0.374
	**p=0.006**	p=0.9	**p=0.01**	p=0.08	p=0.6	**p=0.005**	**p=0.02**

Numbers in bold p-value < 0.05.

Table S2. Baseline, follow-up and expected follow-up RNFL thickness in FRDA patients.

**Patient**	**Baseline RNFL thickness**	**Follow up RNFL thickness**	**Expected Follow Up RNFL thickness ***	**Difference**	**Time from baseline(months)**
2	94	93	93.67	0.33	12
3	114	106.5	113.37	6.87	23
4	81	80	80.175	0.18	30
5	74	73	73.175	0.18	30
6	94	93	93.67	0.33	33
7	108	106	107.37	1.37	23
10	104	90.5	103.37	12.87	23
15	55	44	54.2	10.2	29
18	107	103	105.68	2.68	48
21	81	72.5	80.18	7.68	30
23	74	66	73.2	7.18	30
25	97	95.5	96.18	0.68	30
33	90.5	90	90.12	0.12	14
34	96	94	95.56	1.56	16
36	87	85.5	86.15	0.65	31
37	76	67	75.18	8.18	30
42	83	82.5	82.1	-	33
43	84.5	83	83.73	0.73	28
44	88	86.5	87.23	0.73	28

* Minimal thinning reported in healthy subjects (0.33 μm/year).
